# Biosecuring Animal Populations: The Future Is Now

**DOI:** 10.3390/ani15071048

**Published:** 2025-04-04

**Authors:** Scott Dee, Gordon Spronk

**Affiliations:** Pipestone Research, Pipestone, MN 56164, USA; gordon.spronk@pipestone.com

Dear Colleagues,

It was with great enthusiasm that Dr. Spronk and I agreed to lead this Special Issue on the topic of *Biosecuring Animal Populations*. This was, and still is, a very timely topic, based on the global challenges of domestic and transboundary pathogens of pigs, such as African swine fever virus and porcine reproductive and respiratory syndrome virus, as well as the threat of the One Health crisis secondary to highly pathogenic avian influenza virus across multiple animal species as well as humans ([1,2,3,4]). Looking back, this Special Issue has proven to be multi-disciplinary, involving over 100 authors across 25 countries from 3 continents, and transcends multiple species including pigs, dairy cattle, dairy goats, and poultry, and targets several significant viral pathogens of animals, including porcine reproductive and respiratory syndrome virus, porcine epidemic diarrhea virus, African swine fever virus, classical swine fever virus, Senecavirus A, bovine coronavirus, and hepatitis E virus.

The Special Issue contained 18 new articles that were both educational and diverse, including a Literature Review (Section 1, 1 article), New Research (Section 2, 6 articles), Bio-surveillance (Section 3, 5 articles), Human Behavior and Biosecurity (Section 4, 3 articles) and Industry Action (Section 5, 3 articles). Section 1 opened the Special Issue with a review of biosecurity principles and terminology, providing insight on the science-based practices currently in place to reduce the risk of porcine reproductive and respiratory syndrome virus entry into swine breeding herds in the US (Contribution 1). Section 2, New Research, brought forth 6 exciting new articles, providing new information on technological advancements to measure and enhance compliance of biosecurity protocols, identify “superspreader” herds, evaluated the standardization of biosecurity scoring systems, introducing protocols to improve the ability to decontaminate transport vehicles and feed, reviewing the practices of cleaning and disinfection in swine herds across 10 European countries, as well as describing new advancements in biocontainment to reduce the risk of African swine fever virus escape from controlled facilities (Contributions 3 and 4).

Following these articles, new information on the topic of bio-surveillance is discussed in Section 3, a highly relevant topic in agriculture today. Five articles highlight advancements in the use of pen-based sampling techniques collected from both the population and the environment, describe new approaches to active participatory regional surveillance, and provide updates on assessing the prevalence of novel pathogens, such as bovine coronavirus and hepatitis E virus (Contributions 10 and 11). Section 4 focuses on perhaps the most critical component to the success or failure of a biosecurity program—the role of human behavior in the implementation of known successful interventions at the farm level. Three articles are featured, describing the characteristics of farm personnel, along with a review of the awareness and attitudes of farmers and the role of veterinarians as it pertains to biosecurity. Clearly, due to its importance and complexity, the topic of human behavior and its impact on biosecurity could be a Special Issue on its own! Leadership is needed to cast a vision of successful implementation to change the intentional behavior of farm staff (Contribution 15).

Finally, Section 5 focuses on the field and highlights three novel biosecurity-based initiatives that are underway across the US and Denmark. This is clearly where the “rubber meets the road” as science is put into practice and actual results are measured over time. From Denmark comes a summary of industry actions to mitigate the introduction of African swine fever virus from neighboring European Union countries. The article describes how the Danish pig industry has used science to mitigate the significant risk factors of the spread of African swine fever virus—wild boar, transport, and feed imports (Contribution 16). This is followed by an article which describes experiences from the US Swine Health Improvement Plan (US SHIP), a pilot program based on the National Poultry Improvement Plan that focuses on the prevention of the incursion into the US of African swine fever virus and classical swine fever virus (Contribution 17). The final paper, again from the US, builds on a previous publication describing, for the first time since its emergence more than 35 years ago, the sustainable prevention of the introduction of porcine reproductive and respiratory syndrome virus into breeding herds from a large commercial pork production system following the implementation of science-based biosecurity protocols ([5] and Contribution 18). Truly this is a unique observation that brings hope and optimism to agricultural industries as it pertains to the control and prevention of critical livestock diseases.

In closing, this Special Issue was the perfect opportunity to address a pressing need in agriculture—the biosecuring of animal populations to prevent disease. It brings forth new information which answers several questions, but gaps in the knowledge base still exist at the level of the field, such as: What new technologies will be successfully implemented on-farm? How best to conduct surveillance of large populations? What is the best way to motivate and incentivize farm personnel to properly implement biosecurity plans? Will science-based biosecurity prove to be applicable and sustainable across the industry? Can the global industry successfully curtail the spread of African swine fever virus and what will be the role of US SHIP? Clearly, these are exciting times which require the input and expertise of all veterinary professionals.

Be hopeful,

Scott and Gordon
